# Goepp's Dental State Board Questions and Answers

**Published:** 1912-05-15

**Authors:** 


### BIBLIOGRAPHY.

GOEPP'S DENTAL STATE BOARD QUESTIONS AND ANSWERS. This is a new book of 428 pages, published in good style by the Saunders Company, of Philadelphia. The editor is Professor R. Max Goepp, of the Philadelphia Polyclinic, who is the author of a similar work for medical Examinations, from which a large amount of the material has been taken for the dental work. The questions were gathered from papers submitted at different state examinations, compiled, edited and answered. Of course it would be impossible to make a quiz compend, covering every subject of the dental curriculum, with a comprehensive quiz and reply to all the questions that may be asked. Dr. Goepp has succeeded in giving us a good list of questions and a remarkably appropriate and satisfactory answer to each one. That the book will serve a good purpose in reviewing for a State Board Examination we have no doubt, but we can not see quite how one could remember the answers to so many questions. The book has so many good answers that every practitioner wants to know that it will make a most useful reference book for the practitioner. The price is so reasonable that every one can well afford to have a copy in his library—$2.75 in cloth.

